# Supplementary material for: Investigating the influence of physiologically relevant hydrostatic pressure on CHO cell batch culture
Source: Sci Rep. 2021 Jan 8;11:162. doi: 10.1038/s41598-020-80576-8 (PMC7794228; doi:10.1038/s41598-020-80576-8)
Supplement: Supplementary file 1 — Supplementary Information 1. [file 41598_2020_80576_MOESM1_ESM.zip › S1/IgG/18Dec 4th round 5%CO2/C Dec16.pdf]

Data File C:\Users\P...ents\ChemStation\1\Data\CHO_IgG 2018-12-18 11-44-16\006-4-c Dec18.D
Sample Name: c Dec18

===

Acq. Operator	: SYSTEM	Seq. Line :	6
Sample Operator	: SYSTEM		
Acq. Instrument	: HPLC-0039	Location :	4
Injection Date	: 12/18/2018 12:07:37 PM	Inj :	1
		Inj Volume :	100.000 µl
Sequence File	: C:\Users\Public\Documents\ChemStation\1\Data\CHO_IgG 2018-12-18 11-44-16 \CHO_IgG.S		
Method	: C:\Users\Public\Documents\ChemStation\1\Data\CHO_IgG 2018-12-18 11-44-16 \CHO_IGG_LC.M (Sequence Method)		
Last changed	: 12/18/2018 11:44:20 AM by SYSTEM		

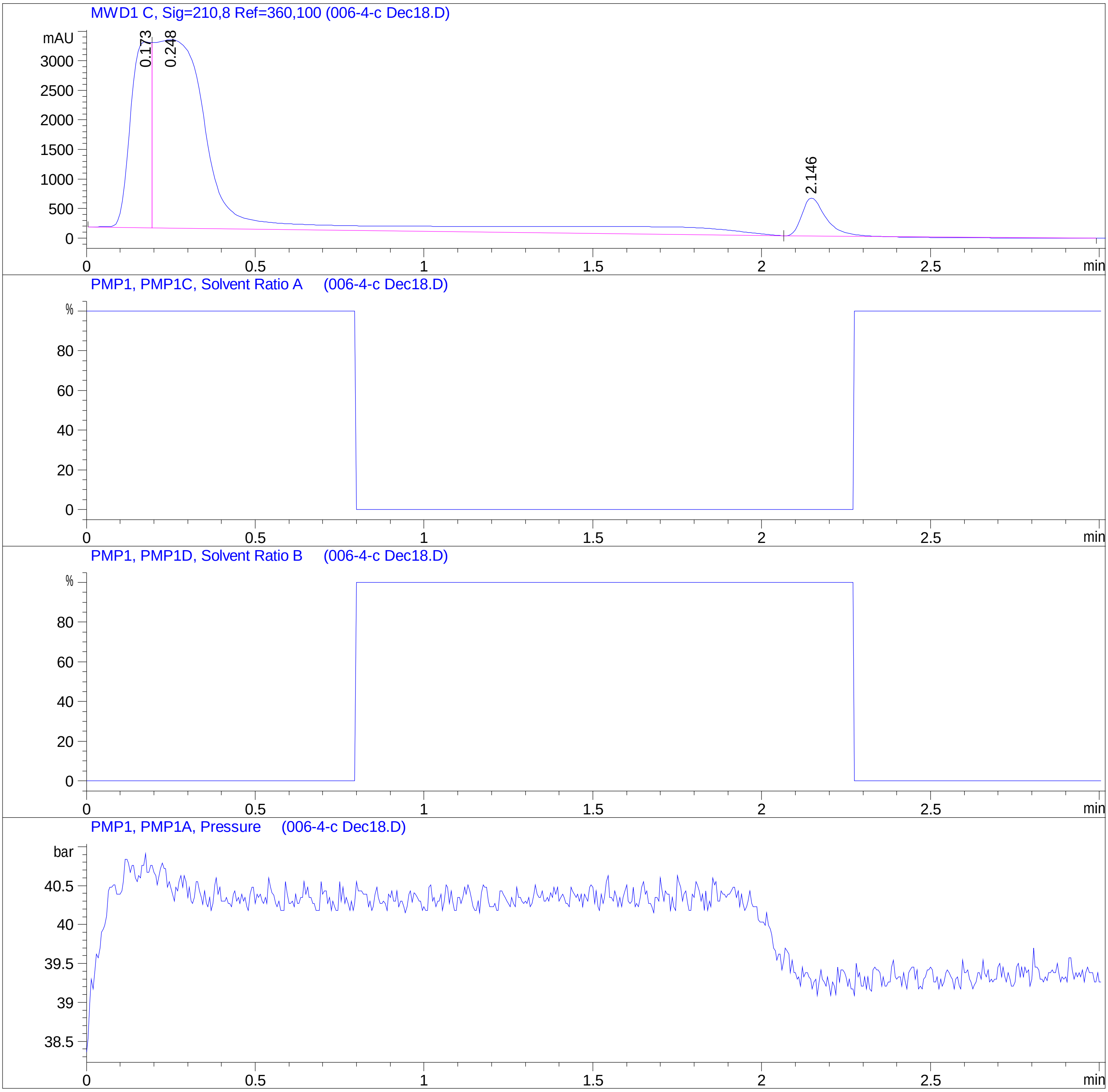

===

Area Percent Report

===

Sorted By : Signal
Multiplier : 1.0000
Dilution : 1.0000
Use Multiplier & Dilution Factor with ISTDs

Signal 1: MWD1 C, Sig=210,8 Ref=360,100

Peak #	RetTime [min]	Type	Width [min]	Area [mAU*s]	Height [mAU]	Area %
1	0.173	BV	0.0682	1.35319e4	3144.46948	23.8880
2	0.248	VV	0.1833	4.02258e4	3185.62866	71.0113
3	2.146	VB	0.0707	2889.39966	639.59204	5.1007

Totals : 5.66471e4 6969.69019

===

*** End of Report ***
